# The relationship between preoperative hypoalbuminemia and postoperative subsyndromal delirium in elderly hip fracture patients: a prospective study

**DOI:** 10.3389/fmed.2025.1631585

**Published:** 2025-12-19

**Authors:** Peipei Li, Zi Ruo Zhang, Dan Chen, Hong Zhi, Jing Hu, Ping Xie, Lian Wen, Si Tong Yan

**Affiliations:** 1 Sports Medicine Diagnosis and Treatment Center, Honghui Hospital, Xi’an Jiaotong University, Xi’an, Shaanxi, China; 2 Foot and Ankle Orthopedic Diagnosis and Treatment Center, Honghui Hospital, Xi’an Jiaotong University, Xi’an, Shaanxi, China; 3 Department of Medicine, Yan’an University, Yan’an, Shaanxi, China; 4 Department of Nursing, Honghui Hospital, Xi’an Jiaotong University, Xi’an, Shaanxi, China; 5 Academic Development Department, Honghui Hospital, Xi’an Jiaotong University, Xi’an, Shaanxi, China; 6 Department of Internal Medicine, Honghui Hospital, Xi’an Jiaotong University, Xi’an, Shaanxi, China

**Keywords:** hip fracture, albumin levels, hypoalbuminemia, SSD, elderly people

## Abstract

**Background:**

This study aimed to assess the prevalence of preoperative hypoalbuminemia in patients with hip fractures at Honghui Hospital, Xi’an Jiaotong University, and to examine its associations with postoperative subsyndromal delirium (SSD) and postoperative discharge-status.

**Methods:**

A prospective study was conducted at Honghui Hospital, Xi’an Jiaotong University, involving patients who underwent hip fracture surgery between October 2023 and March 2024. Data on demographics, comorbidities, preoperative serum albumin levels, SSD occurrence, and postoperative discharge status were also collected. Hypoalbuminemia was defined as serum albumin levels below 35 g/L.

**Results:**

In total, 279 patients were included in the analysis. Hypoalbuminemia was observed in 60 patients (21.51%) and 114 patients (40.86%) exhibited postoperative SSD. Multivariate logistic regression analysis revealed that diabetes mellitus (OR = 2.93, *p* = 0.007), smoking (OR = 4.30, *p* = 0.033), and hypoalbuminemia (OR = 6.13, *p* < 0.001) were independently associated with an elevated risk of SSD. Furthermore, each one-point increase in MMSE score was independently associated with a 1.74 reduction in SSD risk (*p* < 0.001). A threshold effect on the association between albumin levels and SSD was observed when serum albumin level was treated as a continuous variable (*p* for likelihood test = 0.034). Among the 279 patients, 22 were admitted to the ICU; however, further statistical analysis was not conducted for the five patients with hypoalbuminemia who were admitted to the ICU due to low ICU occupancy.

**Conclusion:**

Our study identified a 21.51% prevalence of preoperative hypoalbuminemia in elderly patients undergoing hip fracture surgery, which independently contributed to an increased risk of postoperative SSD. We recommend implementing preoperative interventions to correct hypoalbuminemia.

## Introduction

According to statistics, the annual number of new cases of hip fracture in China exceeds 1 million, and it is on the rise, with a high incidence in the elderly, especially those over 65 years of age, accounting for approximately 95% of the total number (1, 2). Surgery is the primary treatment for hip fractures in the elderly (3). Malnutrition is prevalent in older people with hip fractures and reduces their ability to recover from injuries (4). Markers of malnutrition was low serum albumin and has been found to affect hip fracture outcomes. Bohl et al. (5) study reported that the prevalence of hypoalbuminemia was 45.9% in elderly patients undergoing hip fracture surgery. At the same time, some studies have found that preoperative hypoproteinemia not only significantly affects the immune function and anti-infection ability of patients, but also increases the risk of postoperative complications, including confusion, mortality and prolonged hospital (6–9). In clinical practice, hypoalbuminemia has a serious impact on patients’ quality of life and treatment effects; therefore, in-depth studies are of great clinical significance.

Subsyndromal Delirium (SSD) is characterized by a sudden onset condition affecting attention and consciousness, presenting with one or more core symptoms of delirium but does not meet the criteria for full-blown delirium (4, 10). Hospitalized older patients frequently exhibit signs of acute cognitive decline and attentional impairment, and are ultimately diagnosed with delirium (11). Research indicates that up to 50% of individuals with hip fractures experience episodes of delirium during hospitalized (12). Additionally, certain investigations have revealed that hypoalbuminemia can predict postoperative complications, such as infections, increased mortality rates, and longer hospital stays (13). Hypoalbuminemia is an indicator of malnutrition, reflecting the poor physical condition of patients (14). Hypoalbuminemia is also known to be a risk factor for delirium (15). Nevertheless, studies specifically addressing the influence of low albumin levels on the incidence of subsyndromal delirium following hip fractures in older adults are lacking. Consequently, this research aimed to explore potential risk factors contributing to postoperative subsyndromal delirium in elderly patients with hip fractures and to examine whether low albumin levels are associated with postoperative subsyndromal delirium as well as patient outcomes after surgery.

## Methods

Hospital Ethics Review Board approval was obtained prior to the start of the study (approval number: 202410002). We collected the clinical data of 342 patients who underwent hip fracture between October 2023 and March 2024 at Honghui Hospital, Xi’an Jiaotong University, including patient demographics and preoperative comorbidities such as age, sex, body mass index (BMI), smoking, hemoglobin (Hb) level, hypertension, history of previous cerebrovascular accidents (CVAs), Coronary Heart Disease (CHD), diabetes mellitus (DM), chronic obstructive pulmonary disease (COPD), preoperative creatinine level, preoperative serum albumin level, type of fracture, Mini-Mental State Examination (MMSE) score (16), American Society of Anesthesiologist Physical Status (ASA-PS) score (17), details of the operation such as duration of operation, type of anesthesia, type of operation, depth of anesthesia, and intraoperative blood transfusion (18); postoperative data included the Numeric Rating Scale (NRS) (19), and length of hospital stay (LOS). We defined the window for preoperative serum albumin levels to be taken at admission and a minimum of 1 day before the surgery. We also defined serum albumin levels below 35 g/L as hypoalbuminemia.

The primary outcome was SSD, and the secondary outcomes were postoperative destination, in-hospital ward, or ICU. We used the confusion assessment method (CAM) (20) to collect subsyndromal delirium, which involves four core symptoms (21): ① acute changes in consciousness; ② attention deficits; ③ disorganized thinking; ④ changes in level of consciousness. If one or more of the four core symptoms of CAM are present and do not meet the diagnostic criteria for delirium, it indicates the presence of SSD; if all four core symptoms of CAM are present with characteristic ①, characteristic ② and characteristic ③ (or characteristic ④), it indicates the presence of delirium. Before the study began, the evaluators studied the operation process and scoring criteria of the relevant scales in detail, and learned the operation guide manual of CAM evaluators to ensure the scientificity and reliability of the evaluation work. The CAM assessment was conducted by trained researchers using observation and face-to-face interviews to collect data. If the patient developed symptoms on days 1–3 after surgery, two assessors evaluated the occurrence time, duration (in days), clinical presentation, and whether delirium progressed; if the subject was discharged after surgery, it was considered lost to follow-up; if the subject was transferred to another department after surgery, the investigator will continue to evaluate the subject in the transferred department; if the subject is transferred to the ICU after surgery, the investigator will enter the ICU and evaluate the subject using the same data collection method to reduce the subjective bias of evaluators and improve the objectivity and credibility of the research results. The evaluation of the outcome indicators was conducted by two rigorously trained assessors: the researcher (Assessor A) and another graduate student from the research group (Assessor B). The two assessors stood on opposite sides of the same subject and used a rotating questioning method for blind assessments. In case of disputes, they consulted a neurologist for the final determination, with the physician’s final decision being the standard.

After excluding 38 patients younger than 65 years, 6 patients with combined fractures in the other, 8 patients with more than three missing variables, and 11 patients who did not have preoperative serum albumin levels, we obtained 279 patients in the final analysis.

### Statistical analysis

The collected variables were quality-checked, screened, and sorted, and a database was established using Excel. R4.3.3 software was used for data analysis, and numerical variables were expressed as mean ± standard deviation and categorical variables as frequency and percentages. Data for continuous variables were normally distributed using an independent t-test; data for continuous variables were non-normally distributed using the rank sum test and chi-square test for categorical variables. Logistic regression was used for one-way analysis of each variable, and predictor variables were screened according to the criterion of *p* ≤ 0.05. The factors resulting from screening were included in the multifactorial logistic regression analysis, and a regression model was used to observe the effect of hypoalbuminemia on postoperative subsyndromal delirium in elderly patients with hip fracture under different adjustment strategies. Finally, the serum albumin level was used as a continuous variable for serum albumin and SSD threshold analysis using R4.3.3.

## Results

### Demographics

Of the 279 elderly patients who experienced hip fracture during the study period, 21.51% had hypoalbuminemia, 40.86% had SSD, and hypoalbuminemia 63.33% had SSD. As shown in Table 1. Patients with hypoalbuminemia had lower mean Hb levels than those with normal albumin levels (*p* < 0.001) and lower preoperative creatinine levels (*p* = 0.021). However, patients with hypoalbuminemia tended to have a higher male ASA-PS score (*p* < 0.001), intraoperative blood transfusion (*p* = 0.018), and incidence of SSD (*p* < 0.001). There were no significant differences in the other variables between the two groups.

**Table 1:** Demographics of patients with normal albumin (≥35 g/L) versus those with hypoalbuminemia (<35 g/L) postoperative hip fracture in elderly.

Variables	Total (*n* = 279)	Albumin ≥35 g/L *n* = 219	Albumin <35 g/L *n* = 60	Statistic	*p*
Age, Mean ± SD	75.55 ± 8.01	75.43 ± 8.14	75.97 ± 7.58	*t* = −0.46	0.649
MMSE score, Mean ± SD	22.69 ± 4.76	22.97 ± 4.69	21.67 ± 4.92	*t* = 1.89	0.060
LOS (d), Mean ± SD	9.42 ± 4.34	9.43 ± 4.11	9.37 ± 5.13	*t* = 0.11	0.916
Hb (g/L), Mean ± SD	117.20 ± 20.25	120.66 ± 19.55	104.57 ± 17.68	*t* = 5.76	<0.001
Creatinine (ummol/L), Mean ± SD	67.51 ± 31.52	69.76 ± 33.95	59.07 ± 17.72	*t* = 2.31	0.021
BMI (kg/m^2^), Mean ± SD	22.24 ± 3.46	22.26 ± 3.52	22.18 ± 3.27	*t* = 0.15	0.881
Duration of operation (min), Mean ± SD	110.73 ± 49.60	109.59 ± 45.69	114.90 ± 62.12	*t* = −0.62	0.538
NRS score, M (Q₁, Q₃)	2.00 (1.00, 3.00)	2.00 (1.00, 3.00)	2.00 (1.00, 4.00)	*Z* = –0.74	0.458
Depth of anesthesia, M (Q₁, Q₃)	22.00 (21.00, 22.00)	22.00 (21.00, 22.50)	22.00 (22.00, 22.00)	*Z* = –0.16	0.875
Sex, *n* (%)				*χ*^2^ = 4.92	0.027
Male	114 (40.86)	82 (37.44)	32 (53.33)		
Female	165 (59.14)	137 (62.56)	28 (46.67)		
Type of fracture, *n* (%)				*χ*^2^ = 1.67	0.434
Femoral neck fracture	160 (57.35)	126 (57.53)	34 (56.67)		
Intertrochanteric fracture	101 (36.20)	81 (36.99)	20 (33.33)		
Subtrochanteric fracture	18 (6.45)	12 (5.48)	6 (10.00)		
Hypertension, *n* (%)				*χ*^2^ = 2.95	0.086
No	140 (50.18)	104 (47.49)	36 (60.00)		
Yes	139 (49.82)	115 (52.51)	24 (40.00)		
CHD, *n* (%)				*χ*^2^ = 0.08	0.775
No	229 (82.08)	179 (81.74)	50 (83.33)		
Yes	50 (17.92)	40 (18.26)	10 (16.67)		
DM, *n* (%)				*χ*^2^ = 2.67	0.102
No	210 (75.27)	160 (73.06)	50 (83.33)		
Yes	69 (24.73)	59 (26.94)	10 (16.67)		
CVAs, *n* (%)				*χ*^2^ = 0.68	0.410
No	189 (67.74)	151 (68.95)	38 (63.33)		
Yes	90 (32.26)	68 (31.05)	22 (36.67)		
COPD, *n* (%)				*χ*^2^ = 0.99	0.320
No	265 (94.98)	210 (95.89)	55 (91.67)		
Yes	14 (5.02)	9 (4.11)	5 (8.33)		
Smoking, *n* (%)				*χ*^2^ = 2.03	0.154
No	32 (11.47)	22 (10.05)	10 (16.67)		
Yes	247 (88.53)	197 (89.95)	50 (83.33)		
Type of operation, *n* (%)				*χ*^2^ = 0.37	0.833
THA and partial-hip replacement	154 (55.20)	122 (55.71)	32 (53.33)		
ORIF	12 (4.30)	10 (4.57)	2 (3.33)		
Closed reduction and internal fixation	113 (40.50)	87 (39.73)	26 (43.33)		
ASA-PS score, *n* (%)				–	<0.001
1	4 (1.43)	2 (0.91)	2 (3.33)		
2	92 (32.97)	82 (37.44)	10 (16.67)		
3	181 (64.87)	135 (61.64)	46 (76.67)		
4	2 (0.72)	0 (0.00)	2 (3.33)		
Type of anesthesia, *n* (%)				–	0.107
GA	28 (10.04)	26 (11.87)	2 (3.33)		
RA	2 (0.72)	2 (0.91)	0 (0.00)		
GA + RA	249 (89.25)	191 (87.21)	58 (96.67)		
Intraoperative blood transfusion, *n* (%)				*χ*^2^ = 5.62	0.018
No	176 (63.08)	146 (66.67)	30 (50.00)		
Yes	103 (36.92)	73 (33.33)	30 (50.00)		
SSD, *n* (%)				*χ*^2^ = 15.98	<0.001
No	165 (59.14)	143 (65.30)	22 (36.67)		
Yes	114 (40.86)	76 (34.70)	38 (63.33)		

LOS, length of hospital stay; CHD, coronary heart disease; GA, general anesthesia; RA, regional anesthesia; DM, diabetes mellitus; CVAs, cerebrovascular accidents; COPD, chronic obstructive pulmonary disease; THA, Total Hip Arthroplasty; ORIF, Open reduction and internal fixation; ASA-PS, American Society of Anesthesiologists Physical Status.

### Results of the univariate logistic regression

Univariate logistic regression analysis was performed using SSD as the dependent variable. Based on the univariate analysis presented in Table 2, the variables associated with an elevated risk of SSD included age (OR = 1.06, *p* < 0.001), subtrochanteric fractures (OR = 0.17, *p* = 0.020), DM (OR = 1.79, *p* = 0.036), hypertension (OR = 1.93, *p* = 0.008), CVAs (OR = 1.82, *p* = 0.021), smoking (OR = 3.34, *p* = 0.010), and hypoalbuminemia (OR = 3.25, *p* < 0.001). Additionally, the risk of developing SSD decreases as MMSE scores increase.

**Table 2:** Variables that predict increased SSD postoperative hip fracture in elderly, based on univariate analysis.

Variables	*β*	S.E	*Z*	*p*	OR (95%CI)
Sex					
Male					Reference
Female	0.15	0.25	0.60	0.550	1.16 (0.71–1.89)
Type of fracture					
Femoral neck fracture					Reference
Intertrochanteric fracture	0.03	0.26	0.10	0.919	1.03 (0.62–1.70)
Subtrochanteric fracture	−1.78	0.77	−2.32	0.020	0.17 (0.04–0.76)
CHD					
No					Reference
Yes	0.16	0.32	0.52	0.602	1.18 (0.64–2.19)
DM					
No					Reference
Yes	0.58	0.28	2.09	0.036	1.79 (1.04–3.09)
Hypertension					
No					Reference
Yes	0.66	0.25	2.66	0.008	1.93 (1.19–3.13)
CVAs					
No					Reference
Yes	0.60	0.26	2.31	0.021	1.82 (1.10–3.02)
COPD					
No					Reference
Yes	1.02	0.57	1.78	0.076	2.76 (0.90–8.46)
Smoking					
No					Reference
Yes	1.21	0.47	2.56	0.010	3.34 (1.33–8.41)
Type of operation					
THA and partial-hip replacement					Reference
ORIF	−1.43	0.79	−1.80	0.071	0.24 (0.05–1.13)
Closed reduction and internal fixation	−0.36	0.25	−1.41	0.158	0.70 (0.43–1.15)
ASA-PS score					
1					/
2	15.29	1,199.77	0.01	0.990	/
3	16.63	1,199.77	0.01	0.989	/
4	0.00	2,078.07	0.00	1.000	/
Type of anesthesia					
GA					Reference
RA	−14.28	624.19	−0.02	0.982	0.00 (0.00–Inf)
GA + RA	−0.08	0.40	−0.21	0.834	0.92 (0.42–2.02)
Intraoperative blood transfusion					
No					Reference
Yes	0.23	0.25	0.92	0.358	1.26 (0.77–2.06)
Hypoalbuminemia					
No					Reference
Yes	1.18	0.30	3.89	<0.001	3.25 (1.79–5.89)
Age	0.06	0.02	3.58	<0.001	1.06 (1.03–1.09)
MMSE score	−0.30	0.04	−7.95	<0.001	0.74 (0.69–0.80)
Hb	−0.01	0.01	−1.26	0.207	0.99 (0.98–1.00)
Creatinine	−0.00	0.00	−0.20	0.838	1.00 (0.99–1.01)
BMI	0.01	0.04	0.37	0.708	1.01 (0.95–1.09)
NRS score	−0.05	0.10	−0.51	0.610	0.95 (0.78–1.15)
Depth of anesthesia	−0.22	0.12	−1.88	0.060	0.81 (0.64–1.01)
Duration of operation	−0.00	0.00	−1.33	0.184	1.00 (0.99–1.00)

### Results of the multivariate logistic regression

This study employed a single-factor strategy for variable selection, incorporating variables from Table 2 that demonstrated statistical significance (*p* < 0.05) in univariate analysis into the multivariate regression analysis. Based on the multivariate analysis presented in Table 3, the variables associated with an independent elevated risk of SSD included DM (OR = 2.93, *p* = 0.007), smoking (OR = 4.30, *p* = 0.033), and hypoalbuminemia (OR = 6.13, *p* < 0.001). Each one-point increase in MMSE score was associated with a 26% reduction in the odds of SSD (OR = 0.74, *p* < 0.001).

**Table 3:** Variables that predict increased SSD postoperative hip fracture in elderly, based on multivariate analysis.

Variables	*β*	S.E	*Z*	*p*	OR (95%CI)
Intercept	3.01	2.27	1.33	0.185	20.35 (0.24–1,745.24)
Type of fracture					
Femoral neck fracture					Reference
Intertrochanteric fracture	0.32	0.34	0.96	0.335	1.38 (0.72–2.67)
Subtrochanteric fracture	−1.23	0.86	−1.43	0.153	0.29 (0.05–1.58)
DM					
No					Reference
Yes	1.08	0.40	2.68	0.007	2.93 (1.34–6.44)
Hypertension					
No					Reference
Yes	0.49	0.34	1.46	0.145	1.64 (0.84–3.19)
CVAs					
No					Reference
Yes	0.11	0.35	0.32	0.747	1.12 (0.56–2.23)
Smoking					
No					Reference
Yes	1.46	0.69	2.13	0.033	4.30 (1.12–16.49)
Hypoalbuminemia					
No					Reference
Yes	1.81	0.42	4.28	<0.001	6.13 (2.67–14.05)
Age	0.01	0.02	0.54	0.590	1.01 (0.97–1.06)
MMSE score	−0.29	0.04	−6.63	<0.001	0.74 (0.68–0.81)

In the subgroup analysis stratified by sex (Table 4), males (*n* = 114) showed a moderate association (OR = 2.77, 95% CI: 1.20–6.41, *p* = 0.017), while females (*n* = 166) demonstrated a stronger association (OR = 4.35, 95% CI: 1.79–10.60, *p* = 0.001). However, the interaction effect between sex and albumin levels was not statistically significant (*p* for interaction = 0.469), indicating that sex did not significantly modify the observed association.

**Table 4:** Subgroup analysis based on sex.

Variables	*n* (%)	Albumin ≥35 g/L	Albumin <35 g/L	OR (95%CI)	*p*	*p* for interaction
All patients	279 (100.00)	76/219	38/60	3.25 (1.79–5.89)	<0.001	
Sex						0.469
Male	114 (40.71)	26/82	18/32	2.77 (1.20–6.41)	0.017	
Female	166 (59.29)	50/137	20/28	4.35 (1.79–10.60)	0.001	

### Albumin and SSD threshold analysis results

**Figure 1:**
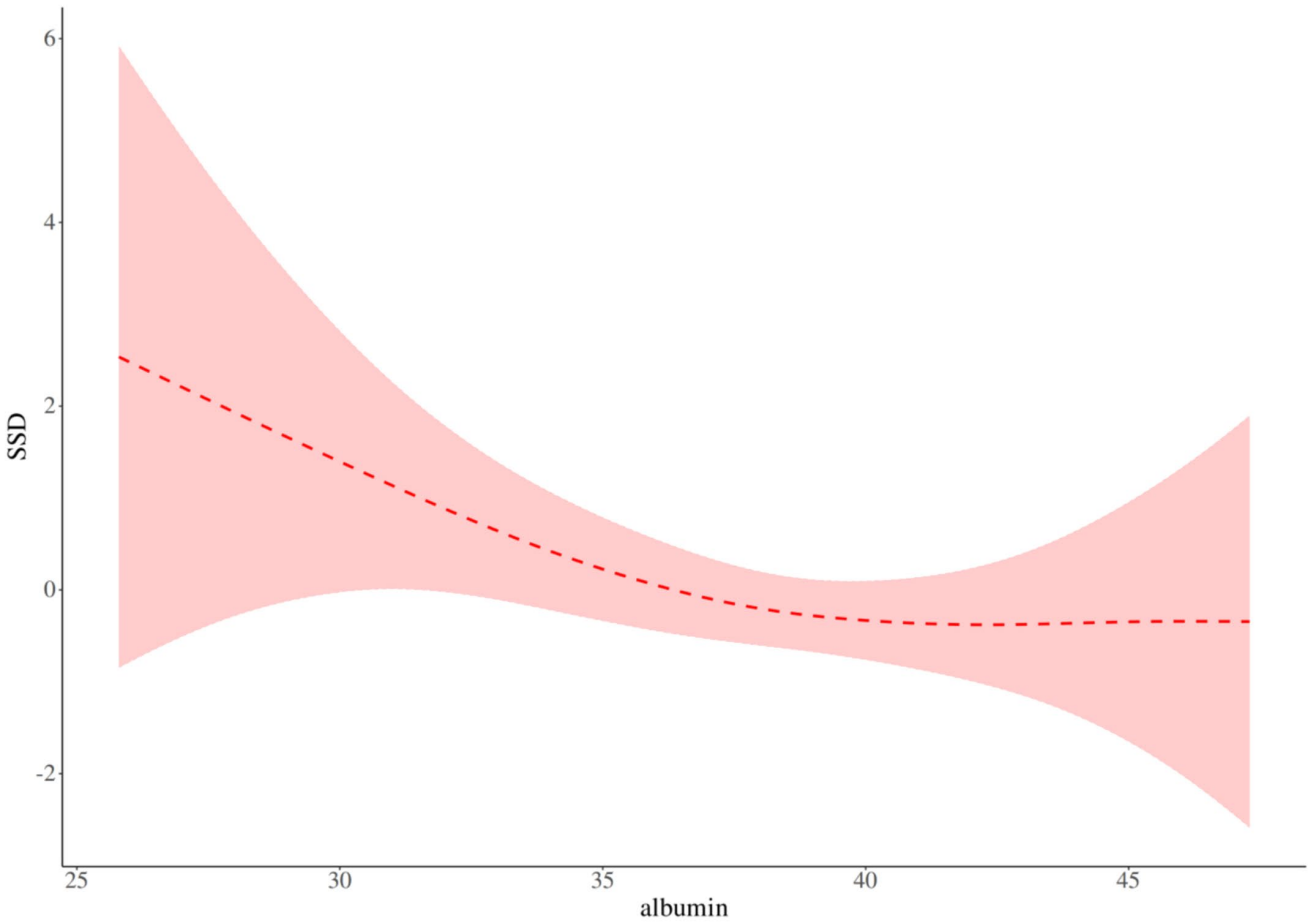
Albumin and SSD threshold analysis.

**Figure 2:**
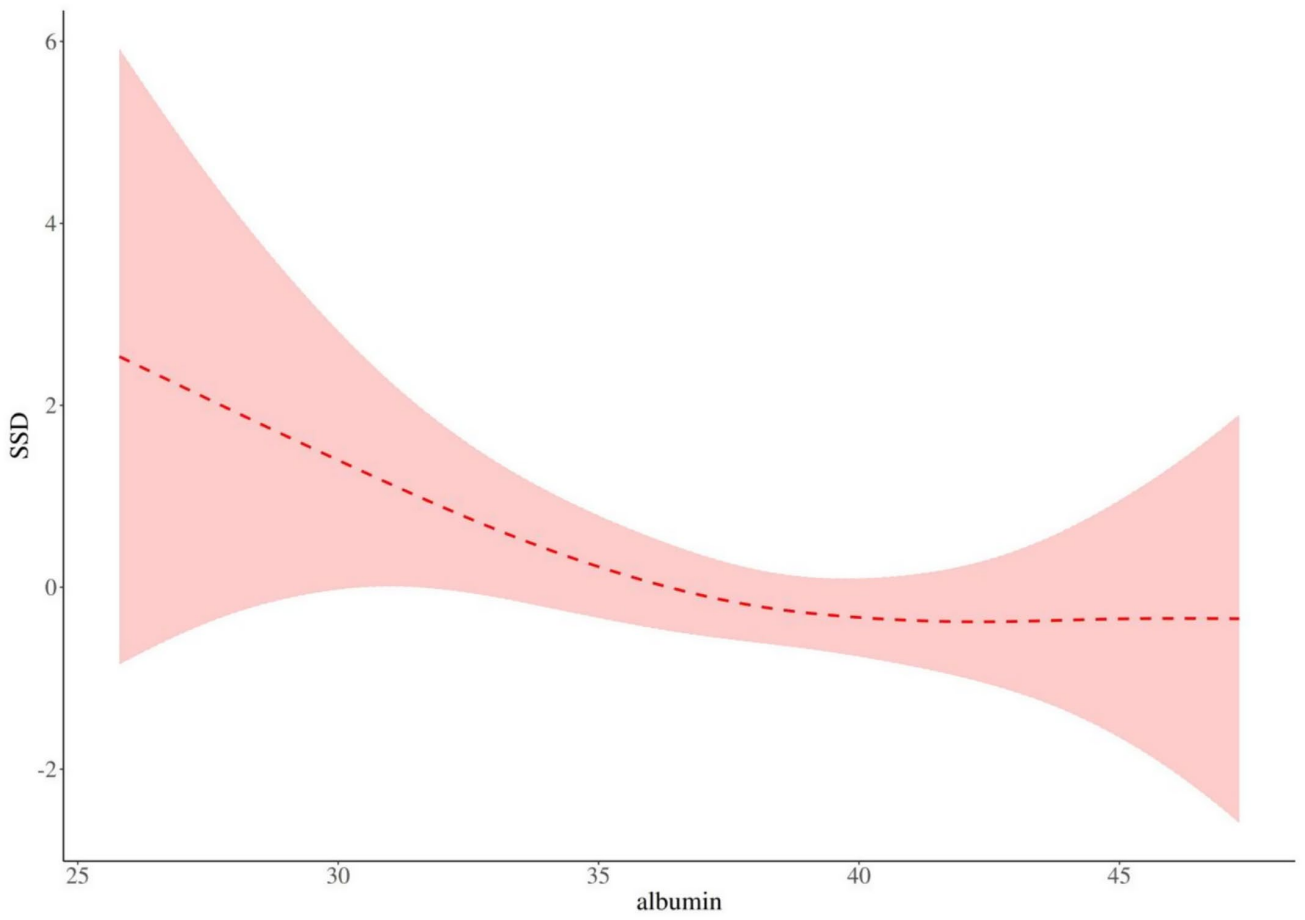
Albumin and SSD threshold analysis after adjustment for covariates.

As shown in Table 5 and Figure 1, there is a threshold effect for the association of albumin and SSD (*p* for likelihood test = 0.034); in general, albumin and SSD were negatively associated [OR (95%CI): 0.89 (0.83–0.95), *p* < 0.001], when the albumin is below 38.4 g/L, albumin and SSD were negatively associated [OR (95%CI): 0.78 (0.69–0.90), *p* < 0.001]; when the albumin level was above 38.4 g/L, no association of albumin and SSD was found; at the same time, I also adjusted for sex, age, intraoperative blood transfusion, and Hb, and the results showed that there is a threshold effect for the association of albumin and SSD (*p* for likelihood test = 0.025), albumin, and SSD were negatively associated [OR (95%CI): 0.87 (0.80–0.94), *p* < 0.001], Figure 2.

**Table 5:** Threshold analysis results.

Outcome	Effect	*p*
Model 1 Fitting model by standard linear regression	0.89 (0.83–0.95)	<0.001
Model 2 Fitting model by two-piecewise linear regression		
Inflection point	38.4	
<38.4	0.78 (0.69–0.90)	<0.001
≥38.4	1.03 (0.87–1.22)	0.713
*p* for likelihood test		0.034

### Postoperative destination

A total of 22 patients went to the ICU among the 279 patients (Table 6). Five patients with hypoalbuminemia were admitted to the ICU, and no further statistical analysis was performed owing to the low ICU occupancy rate.

**Table 6:** Postoperative destination of hip fracture in elderly.

Variables	Total *n* = 279	Albumin ≥35 g/L *n* = 219	Albumin <35 g/L *n* = 60
Postoperative destination, *n* (%)			
In-hospital ward	257 (92.11)	202 (92.24)	55 (91.67)
ICU	22 (7.89)	17 (7.76)	5 (8.33)

## Discussion

Our study found that the incidence of preoperative hypoalbuminemia in elderly patients undergoing hip fracture surgery was 21.51%, whereas the incidence of SSD in patients with hypoalbuminemia was 63.33%. Although the classical definition of hypoalbuminemia is <30 g/L, some studies have reported that serum total protein is <60 g/L or albumin is <35 g/L as hypoalbuminemia, because even mild hypoalbuminemia affects the outcome of patients (22, 23). The criterion for hypoalbuminemia in this study was an albumin level of <35 g/L. The incidence of preoperative hypoalbuminemia is not low, with an incidence of 14–20.7% (24, 25). Our study showed a higher incidence of hypoalbuminemia than that reported in the literature. The first studied population was older and often had a certain degree of digestive and absorption function, insufficient intake of protein and other nutrients, and limited absorption, resulting in varying degrees of preoperative malnutrition (26). Second, hip fractures in the elderly are mostly osteoporotic fractures, and recent studies have found a correlation between hypoalbuminemia and osteoporosis and fracture, which may affect bone metabolism in various ways, resulting in decreased bone mass, decreased bone density, and osteoporosis (27). Afshinnia and Pennathur (28) found that the incidence of osteoporosis in the femoral neck in patients with hypoalbuminemia was significantly higher than that in those with normal albumin, and the difference was statistically significant; therefore, hypoalbuminemia should be actively corrected to reduce the occurrence of related osteoporosis, reduce muscle strength reduction, and prevent the occurrence of osteoporotic fractures the same time. Most studies have reported a significant relationship between hypoalbuminemia and poor patient prognosis (29, 30), the incidence of SSD in hypoalbuminemia has rarely been reported.

Our study found that subjects with hypertension had a higher risk of developing SSD compared to healthy subjects (*p* < 0.001). Hypoalbuminemia was an independent influencing factor affecting postoperative SSD in elderly patients with hip fracture, consistent with previous studies that showed that the preoperative risk of malnutrition and malnutrition status increased the risk of postoperative delirium (31). Albumin is an important indicator of the nutritional status of the body and plays an important role in the overall function of the body (32). The relationship between nutritional status and rehabilitation after fracture has been reported in some studies, including hip function after hip fracture (7) and the risk of postoperative SSD (33). Therefore, comprehensive nutritional assessment should be conducted in elderly patients with hip fracture, focusing attention on patients with albumin below 35 g/L, and preoperative albumin as a key measurement index to accurately assess the risk of postoperative SSD.

Our study also found that individuals with diabetes had a higher risk of SSD than those without diabetes (*p* = 0.007). Consistent with the results of a study by Kalyan et al., elderly patients with diabetes and other complications were more likely to develop SSD (34). In the study, smokers were found to have a higher risk of developing SSD (*p* = 0.033). However, there have been few studies addressing the link between smoking and SSD. Moreover, each one-point increase in MMSE score was associated with a 26% reduction in the odds of SSD. Consistent with the findings of Denny and Such (35), MMSE is an independent risk factor for postoperative SSD in older adults, highlighting preoperative diabetes and poor preoperative cognitive function as risk factors for high risk of SSD, thus using screening for diabetes and cognitive function assessment as a routine preoperative examination item for patients with hip fracture over 65 years of age.

Our results showed a threshold effect on the association of albumin and SSD (*p* for likelihood test = 0.034), and when albumin<38.4 g/L, the decrease in SSD threshold after increased albumin levels was statistically significant (*p* < 0.05). However, at albumin≥38.4 g/L, no association of albumin and SSD was found. The results of the study by Lu et al. (36) showed that Preoperative albumin were associated with postoperative delirium, potentially aiding in identifying high-risk patients and playing a key role in preventing POD. The study was shown by Wang et al. (37) treating preoperative serum albumin level as a continuous variable, the risk of postoperative (POD) delirium increased by 11% (95%CI, 1.08–1.15) with each 1 g/L decrease in preoperative serum albumin level. It was also found that maintaining preoperative serum albumin levels above 38 g/L may lead to a more favorable outcome. Probably because, in the central nervous system, low serum albumin levels appear to be insufficient in fully exerting their antioxidant effects and capturing free radicals (38–40). Normal serum albumin levels can provide essential amino acids to the cell and immune system, thereby optimizing the composition ratio between albumin and other inflammatory factors to enhance the body’s immune response capabilities (41, 42). Furthermore, the evaluation of preoperative serum albumin levels could facilitate early identification of high-risk SSD. Therefore, the aim of our study was to encourage clinical healthcare professionals to re-emphasize monitoring and management of preoperative serum albumin levels in elderly patients with hip fractures.

One of the aims of our study was to identify the risk factors for postoperative destination in our study population. However, out of 279 patients, a total of 22 patients went to the ICU, and 5 of patients with hypoproteinemia went to the ICU with low ICU occupancy without further statistical analysis.

### Strengths of study

We focused our study on elderly patients who underwent hip fracture, which is more commonly performed in the literature, as we wanted a more homogenous study population. The outcome of our study focused on the occurrence of SSD rather than delirium, and the identification of SSD helped improve the vigilance of the prestate of medical staff and reduce missed diagnoses and misdiagnoses due to inconspicuous symptoms.

### Study limitations

This study was limited by its single-center design, potentially impacting the sample size and population homogeneity, which could introduce selection bias during data analysis. Additionally, the rapid changes in hypoalbuminemia over a short period may pose challenges in clinical application, potentially diminishing the significance of hypoalbuminemia as a modifiable risk factor. Moreover, SSD is a multifaceted condition that cannot be solely attributed to a single risk factor, necessitating a comprehensive management approach to effectively mitigate SSD occurrence. At the same time, CAM features may overestimate the incidence of SSD. In later studies, we may use more detailed criteria. Finally, due to limitations in human and material resources, we only analyzed the relationship between preoperative albumin levels and SSD. We did not further examine the relationship between postoperative albumin levels and SSD. Further investigation is needed in the future.

## Conclusion

In conclusion, our study revealed a 21.51% prevalence of preoperative hypoalbuminemia among elderly patients undergoing hip fracture surgery at our institution, establishing it as an independent risk factor for postoperative SSD. We advocate for preoperative interventions to address hypoalbuminemia.

## Data Availability

The original contributions presented in the study are included in the article/Supplementary material, further inquiries can be directed to the corresponding authors.

## References

[1] Zhang C Feng J Wang S Gao P Xu L Zhu J . Incidence of and trends in hipfracture among adults in urban China: a nationwide retrospectivecohort study. PLoS Med. (2020) 17:e1003180. doi: 10.1371/journal.pmed.1003180, 32760065 PMC7410202

[2] Griffiths R Babu S Dixon P Freeman N Hurford D Kelleher E . Guideline for the management of hip fractures 2020: guideline by the association of anaesthetists. Anaesthesia. (2021) 76:225–37. doi: 10.1111/anae.15291, 33289066

[3] Sing CW Lin TC Bartholomew S Bell JS Bennett C Beyene K . Global epidemiology of hip fractures: secular trends in incidence rate, post-fracture treatment, and all-cause mortality. J Bone Miner Res. (2023) 38:1064–75. doi: 10.1002/jbmr.4821, 37118993

[4] Gao Y Gan XN Yang RQ . Conceptual connotation and research progress of subsyndromal delirium. Chin J Nurs. (2021) 56:1883–8.

[5] Bohl DD Shen MR Hannon CP Fillingham YA Darrith B Della Valle CJ. Serum albumin predicts survival and postoperative course following surgery for geriatric hip fracture. J Bone Joint Surg Am. (2017) 99:2110–8. doi: 10.2106/JBJS.16.01620, 29257017

[6] Takimoto M Yasui-Yamada S Nasu N Kagiya N Aotani N Kurokawa Y . Development and validation of cutoff value for reduced muscle mass for GLIM criteria in patients with gastrointestinal and hepatobiliary-pancreatic cancers. Nutrients. (2022) 14:943. doi: 10.3390/nu14050943, 35267918 PMC8912591

[7] Malafarina V Reginster JY Cabrerizo S Bruyère O Kanis JA Martinez JA . Nutritional status and nutritional treatment are related to outcomes and mortality in older adults with hip fracture. Nutrients. (2018) 10:555. doi: 10.3390/nu10050555, 29710860 PMC5986435

[8] Abd-El-Aziz MA Hübner M Demartines N Larson DW Grass F . Simple clinical screening underestimates malnutrition in surgical patients with inflammatory bowel disease-an ACS NSQIP analysis. Nutrients. (2022) 14:932. doi: 10.3390/nu14050932, 35267906 PMC8912602

[9] Sofić A Rašić I Halilović E Mujić A Muslić D . Is Preoperative hypoproteinemia associated with colorectal cancer stage and postoperative complications? Med Glas (Zenica). (2021) 18:450–5. doi: 10.17392/1353-21, 34190507

[10] Denny D Trotter T Lindseth G. Preoperative nutritional status and subsyndromal delirium in older adults following joint replacement surgery. Orthop Nurs. (2020) 39:384–92. doi: 10.1097/NOR.0000000000000710, 33234908

[11] Mailhot T Darling C Ela J Malyuta Y Inouye SK Saczynski J. Family identification of delirium in the emergency department in patients with and without dementia: validity of the family confusion assessment method (FAM-CAM). J Am Geriatr Soc. (2020) 68:983–90. doi: 10.1111/jgs.16438, 32274799 PMC7370702

[12] Inouye SK Westendorp RG Saczynski JS. Delirium in elderly people. Lancet. (2014) 383:911–22. doi: 10.1016/S0140-6736(13)60688-1, 23992774 PMC4120864

[13] Jin J Xiong G Wang X Peng F Zhu F Wang M . The impact of preoperative and postoperative malnutrition on outcomes for ampullary carcinoma after pancreaticoduodenectomy. Front Oncol. (2021) 11:748341. doi: 10.3389/fonc.2021.748341, 34917503 PMC8669645

[14] Wu D Wang X Shi G Sun H Ge G. Prognostic and clinical significance of modified Glasgow prognostic score in pancreatic cancer: a meta-analysis of 4,629 patients. Aging (Albany NY). (2021) 13:1410–21. doi: 10.18632/aging.202357, 33406501 PMC7835027

[15] van der Wulp K van Wely M van Heijningen L van Bakel B Schoon Y Verkroost M . Delirium after transcatheter aortic valve implantation under general anesthesia: incidence, predictors, and relation to long-term survival. J Am Geriatr Soc. (2019) 67:2325–30. doi: 10.1111/jgs.16087, 31342524 PMC6899857

[16] Zhou X Xie M Tao J . Research and application of the simple intelligent mental state examination scale. Chin J Rehabil Med. (2016) 31:694–696+706.

[17] Mupparapu M Singer SR. Editorial: the American Society of Anesthesiologists (ASA) physical status classification system and its utilization for dental patient evaluation. Quintessence Int. (2018) 49:255–6. doi: 10.3290/j.qi.a40053, 29532816

[18] Newman JM Szubski CR Barsoum WK Higuera CA Molloy RM Murray TG. Day of surgery affects length of stay and charges in primary total hip and knee arthroplasty. J Arthroplast. (2017) 32:11–5. doi: 10.1016/j.arth.2016.06.032, 27471211

[19] Gagliese L Weizblit N Ellis W Chan VWS. The measurement of postoperative pain: a comparison of intensity scales in younger and older surgical patients. Pain. (2005) 117:412–20. doi: 10.1016/j.pain.2005.07.004, 16153776

[20] Inouye SK van Dyck CH Alessi CA Balkin S Siegal AP Horwitz RI. Clarifying confusion: the confusion assessment method. A new method for detection of delirium. Ann Intern Med. (1990) 113:941–8. doi: 10.7326/0003-4819-113-12-941, 2240918

[21] Rudolph JL Harrington MB Lucatorto MA Chester GF Francis J . Validation of a medical record-based delirium risk assessment. J Am Geriatr Soc. (2011) 59:S289–94. doi: 10.1111/j.1532-5415.2011.03677.x 22091575 PMC4880478

[22] Truong A Hanna MH Moghadamyeghaneh Z Stamoseh MJ. Implications of preoperative hypoalbuminemia in colorectal surgery. World J Gastrointest Surg. (2016) 8:353–62. doi: 10.4240/wjgs.v8.i5.353, 27231513 PMC4872063

[23] Hu WH Eisenstein S Parry L Ramamoorthy S. Preoperativemal nutrition with mild hypoalbuminemia associated with postoperative mortality and morbidity of colorectal cancer: a propensity score matching study. Nutr J. (2019) 18:33. doi: 10.1186/s12937-019-0458-y, 31253199 PMC6598281

[24] Garth AK Newsome CM Simmance N Crowe TC. Nutritional status, nutrition practices and post-operative complications in patients with gastrointestinal cancer. J Hum Nutr Diet. (2010) 23:393–401. doi: 10.1111/j.1365-277X.2010.01058.x, 20337847

[25] Lai CC You JF Yeh CY Chen JS Tang R Wang JY . Low preoperative serum albumin in colon cancer: a risk factor for poor outcome. Int J Color Dis. (2011) 26:473–81. doi: 10.1007/s00384-010-1113-4, 21190025

[26] Nishimura Y Inagaki Y Noda T Nishioka Y Myojin T Ogawa M . Risk factors for mortality after hip fracture surgery in Japan using the national database of health insurance claims and specific health checkups of Japan. Arch Osteoporos. (2023) 18:91. doi: 10.1007/s11657-023-01293-z, 37418095 PMC10329059

[27] Afshinnia F Pennathur S. Association of hypoalbuminemia with osteoporosis: analysis of the national health and nutrition examination survey. J Clin Endocrinol Metab. (2016) 101:2468–74. doi: 10.1210/jc.2016-1099, 27144935 PMC4891797

[28] Afshinnia F Wong KK Sundaram B Ackermann RJ Pennathur S. Hypoalbuminemia and osteoporosis: reappraisal of a controversy. J Clin Endocrinol Metab. (2016) 101:167–75. doi: 10.1210/jc.2015-3212, 26600169 PMC4701840

[29] Arques S. Serum albumin and cardiovascular disease: does low serum albumin contribute to the emergence and worsening of some cardiovascular diseases? Eur J Intern Med. (2020) 80:122–3. doi: 10.1016/j.ejim.2020.07.019, 32732171

[30] Mitchell P Thomas B Nandan A . Association between albumin level and mortality among cardiac intensive care unit patients. J Intensive Care Med. (2021) 36:1475–82. doi: 10.1177/0885066620963875 33016174

[31] Mazzola P Ward L Zazzetta S Broggini V Anzuini A Valcarcel B . Association between preoperative malnutrition and postoperative delirium after hip fracture surgery in older adults. J Am Geriatr Soc. (2017) 65:1222–8. doi: 10.1111/jgs.14764, 28263371 PMC6555399

[32] Zhang S Liu L Yang B Li R Luo J Huang J . Clinical characteristics of 134 convalescent patients with COVID-19 in Guizhou, China. Respir Res. (2020) 21:314. doi: 10.1186/s12931-020-01580-0, 33243228 PMC7689638

[33] Li J Feng Y Yang F. Analysis of subsyndrome incidence and influencing factors in surgical ICU patients. Chin J Mod Nurs. (2019) 25:1786–90.

[34] Kalyan P Parulekar M. Impact of the Charlson comorbidity index on delirium outcomes. Cureus. (2024) 16:e70006. doi: 10.7759/cureus.70006, 39445285 PMC11498349

[35] Denny DL Such TL. Exploration of relationships between postoperative pain and subsyndromal delirium in older adults. Nurs Res. (2018) 67:421–9. doi: 10.1097/NNR.0000000000000305, 30067582

[36] Lu Z Wang B Liu M Yu D Li J. Correlation analysis between plasma biomarkers albumin, fibrinogen, and their ratio with postoperative delirium in patients undergoing non-cardiac surgery: a systematic review and meta-analysis. Am J Transl Res. (2024) 16:363–73. doi: 10.62347/AEHR2759, 38463596 PMC10918125

[37] Wang W Yao W Tang W Li Y Lv Q Ding W. Association between preoperative albumin levels and postoperative delirium in geriatric hip fracture patients. Front Med (Lausanne). (2024) 11:1344904. doi: 10.3389/fmed.2024.1344904, 38420358 PMC10899384

[38] Venkatakrishnaiah NK Anandkumar UM Wooly S Rajkamal G Gadiyar HB Janakiraman P . Identification of factors contributing to the development of postoperative delirium in geriatric patients with hip fractures-a prospective study. J Family Med Prim Care. (2022) 11:4785–90. doi: 10.4103/jfmpc.jfmpc_238_22, 36353041 PMC9638675

[39] Li S Zhang J Zheng H Wang X Liu Z Sun T. Prognostic role of serum albumin, total lymphocyte count, and mini nutritional assessment on outcomes after geriatric hip fracture surgery: a meta-analysis and systematic review. J Arthroplast. (2019) 34:1287–96. doi: 10.1016/j.arth.2019.02.003, 30852065

[40] Qi J Liu C Chen L Chen J. Postoperative serum albumin decrease independently predicts delirium in the elderly subjects after total joint arthroplasty. Curr Pharm Des. (2020) 26:386–94. doi: 10.2174/1381612826666191227153150, 31880243

[41] Palm W Thompson CB. Nutrient acquisition strategies of mammalian cells. Nature. (2017) 546:234–42. doi: 10.1038/nature22379, 28593971 PMC5541675

[42] Jonas O Keibler MA Davidson SM Keibler MA Hou HW Luengo A . Direct evidence for cancer-cell-autonomous extracellular protein catabolism in pancreatic tumors. Nat Med. (2017) 23:235–41. doi: 10.1038/nm.4256, 28024083 PMC5407288

